# “Methodological Constraints and Interpretive Overreach in ESR1–Breast Cancer Association Studies: A Critical Appraisal”

**DOI:** 10.1002/hsr2.71922

**Published:** 2026-02-28

**Authors:** Ibadullah Tahir, Hunain Shahbaz

**Affiliations:** ^1^ Ayub Medical College Abbottabad Pakistan

Dear Editor,

We read with interest the recent article by Hossen examining associations between ESR1 polymorphisms (rs2234693 and rs9340799) and breast cancer risk in Bangladeshi women [1]. While the study addresses an underrepresented population, several methodological and interpretative limitations substantially weaken the validity and translational relevance of its conclusions.

The modest number of cases and controls raises significant concerns about statistical power for this study. Genetic association studies tend to find small effect sizes and, therefore, require much greater numbers of subjects than those employed in this study, to avoid generating false‐positive results [2]. Furthermore, since there were no power calculations conducted and no independent replication cohort available, confidence in these reported associations is further compromised. Additionally, although population stratification is known to be a confounding variable in South Asian genetic studies, this variable was neither addressed nor adjusted in the course of this study, contrary to current STREGA guidelines [3].

Second, the analytical strategy is problematic. Multiple genetic models and extensive subgroup analyses were performed without correction for multiple testing, markedly inflating Type I error risk. Notably, the statistically “significant” associations for rs2234693 consistently demonstrate odds ratios below unity, indicating a protective rather than susceptibility effect, yet the findings are repeatedly interpreted as increased breast cancer risk [1]. This misinterpretation of effect direction represents a critical conceptual flaw.

Thirdly, the highly restricted examination of just two intronic ESR1 SNPs represents a “quasi genic” or old school reductionist paradigm. Breast cancer is recognized as a complex disease. The use of single‐gene analyses to inform clinical decision‐making is limited and does not provide significant utility in terms of the explanatory power available. Rather, new research has shown that the integrated use of multi‐gene analytical techniques, for example, multigene panels or polygenic risk scores have far greater value than single SNP analyses for predicting cancer risk and generating more effective clinical risk stratification than isolating individual candidate SNPs [4]. Without including additional genetic loci associated with predisposition and/or other multi‐nuclear pathways related to the development of Breast Cancer, the clinical utility of results would be minimal.

Ultimately, there was no functional validation performed to confirm biological plausibility for these intronic variants, as there has been no experimental evidence provided to indicate that they can alter splicing or transcriptional regulation. Studies using genomic methods have previously demonstrated that combining sequencing data with functional assays is a vital method for identifying causal variants rather than statistical “noise” [5].

Overall, despite providing population‐specific information to start understanding the genetic contributions to the development of breast cancer, this study has limited statistical power and lack of functional validation, nor was it designed to fully explore how a provider might recommend testing in cases where a family history is absent. Future studies will need to have sufficient sample sizes with better statistical designs, consider appropriate multiple testing corrections, incorporate data from multiple genes or genome‐wide associations, and include functional studies or multiomic data sets to advance the field of precision oncology in breast cancer.

## Author Contributions

**Ibadullah Tahir:** conceptualization, investigation, funding acquisition, writing – original draft, methodology, validation, visualization, writing – review and editing, project administration, formal analysis, software, data curation, supervision, resources. **Hunain Shahbaz:** funding acquisition and investigation. All authors have read and approved the final version of manuscript.

## Funding

The authors received no specific funding for this work.

## Ethics Statement

The authors have nothing to report.

## Consent

The authors have nothing to report.

## Conflicts of Interest

The authors declare no conflicts of interest.

## Data Availability

The authors have nothing to report.
